# The Importance of the Regional Species Pool, Ecological Species Traits and Local Habitat Conditions for the Colonization of Restored River Reaches by Fish

**DOI:** 10.1371/journal.pone.0084741

**Published:** 2014-01-03

**Authors:** Stefan Stoll, Jochem Kail, Armin W. Lorenz, Andrea Sundermann, Peter Haase

**Affiliations:** 1 Department of River Ecology and Conservation, Senckenberg Research Institute and Natural History Museum, Gelnhausen, Germany; 2 Biodiversity and Climate Research Centre, Frankfurt/Main, Germany; 3 Department of Biology and Ecology of Fishes, Leibniz-Institut for Freshwater Ecology and Inland Fisheries, Berlin, Germany; 4 Department of Aquatic Ecology, University of Duisburg-Essen, Essen, Germany; James Cook University, Australia

## Abstract

It is commonly assumed that the colonization of restored river reaches by fish depends on the regional species pools; however, quantifications of the relationship between the composition of the regional species pool and restoration outcome are lacking. We analyzed data from 18 German river restoration projects and adjacent river reaches constituting the regional species pools of the restored reaches. We found that the ability of statistical models to describe the fish assemblages established in the restored reaches was greater when these models were based on ‘biotic’ variables relating to the regional species pool and the ecological traits of species rather than on ‘abiotic’ variables relating to the hydromorphological habitat structure of the restored habitats and descriptors of the restoration projects. For species presence in restored reaches, ‘biotic’ variables explained 34% of variability, with the occurrence rate of a species in the regional species pool being the most important variable, while ’abiotic’ variables explained only the negligible amount of 2% of variability. For fish density in restored reaches, about twice the amount of variability was explained by ‘biotic’ (38%) compared to ‘abiotic’ (21%) variables, with species density in the regional species pool being most important. These results indicate that the colonization of restored river reaches by fish is largely determined by the assemblages in the surrounding species pool. Knowledge of species presence and abundance in the regional species pool can be used to estimate the likelihood of fish species becoming established in restored reaches.

## Introduction

In recent years, a large amount of evidence has been gathered indicating that reach-scale river restoration projects often fail to meet their predefined goals and, in particular, that reach-scale restoration of the local hydromorphological conditions often does not lead to the re-establishment of natural communities [1]–[6]. Based on this rapidly expanding body of published case studies, Bernhardt and Palmer [7] noted that river restoration research should progress to identifying the drivers that determine the success or failure of restoration projects. To date, very few attempts have been made to integrate the results of multiple river restoration projects and determine relevant variables for restoration outcomes. However, a sound evaluation of different methods and measures is necessary to increase the success of future restorations [8], [9].

In the few comparative studies that exist, the effects of restoration on the species assemblage was primarily related to ‘abiotic’ variables, such as the restoration measures that were carried out, or the hydromorphological quality of the newly created habitats [10]–[14]. These studies found it difficult to identify variables that were conclusively associated with restoration success [11]. Analysis on the outcome of 13 lowland river restoration projects in which flow deflectors and artificial riffles were installed showed inconsistent effects on fish assemblages in terms of species richness, diversity and equitability [12]. Also the effectiveness of wood placement for enhancing fish assemblages varied between individual projects [13].

In addition to the potential effects of ‘abiotic’ variables on the outcome of river restoration projects, the ‘biotic’ components that may determine whether river restorations meet their goals have received increasing attention [15]. In this context, some authors focused on the role of the regional species pool [16], [17]. Applying a filter model [18], local assemblages in restored sites may be forecasted from the regional species pools based on species dispersal capabilities, and local hydromorphological and ‘biotic’ constraints [19], [20]. In particular, dispersal should play a vital role in structuring the regional population networks within the dendritic structure of a river system [21]. A recent study on the spatial extent of the species pool available for the colonization of restored reaches in streams and rivers in Central Europe found that 96.6% of the fish species recorded in restored reaches had nearby source populations within a range of 5 km around the restored reach [10]. Species with their closest source populations further away than 5 km rarely colonized restored reaches.

Where dispersal is not artificially limited, the probability that a restored reach will be colonized by a fish species should depend on the species population structure within the regional species pool [16], [22]. Thereby the colonization of a restored site is largely determined by the propagule rain it receives, which in turn is affected by the number and size of potential source populations within a critical dispersal distance [22], [23]. To date, the role of the population structure in the species pool on the outcome of restoration projects is largely unknown.

In addition to quantitative characteristics of the regional species pool, a species’ ability to colonize a restored reach will be determined by its ecological traits. In the context of dispersal and colonization, in particular morphological traits that potentially affect the mobility of species are important [24], [25]. Also other traits like habitat preference and foraging type may be indirectly related to mobility and the ability of species to establish at restored reaches. Furthermore taxonomic affiliation has been shown to be a good indicator for the dispersal abilities of fish [24]. In this study, these variables on population structure and species traits are summed up as ‘biotic’ variables to contrast them to ‘abiotic’ variables characterizing the hydromorphological quality of the restored reaches.

To gain a better understanding of the key variables that are best able to explain species assemblages at restored river reaches, we applied separate statistical models to explain the fish assemblages at restored reaches using ‘biotic’ and ‘abiotic’ variables and compared the proportion of variability that they account for. We used data from 18 river restoration projects and associated species pools from Germany to address the following questions: (1) How well do ‘biotic’ variables relating to the regional species pool explain the fish assemblages that colonise restored reaches? (2) Are these ‘biotic’ variables better suited to predict fish assemblages in restored reaches than ‘abiotic’ hydromorphological habitat characteristics? This information is highly relevant for restoration managers, as it can be used for a realistic and target-oriented approach to river restoration, and thus can help to increase river restoration effectiveness.

## Materials and Methods

### Ethics Statement

All animal work has been conducted in accordance with relevant national and international guidelines.

### Sampling Sites

We investigated 18 reach-scale river restoration projects in third- to seventh-order rivers in the low-elevation mountain ranges of the German federal states of Hesse and North Rhine-Westphalia. The goal of these projects was to restore the animal and plant communities to a more natural state by improving and diversifying the hydromorphological structure of the rivers (Table 1). To achieve this overarching restoration goal, a variety of measures were applied, including the removal of bank fixations, the creation of a more braided and meandering planform and the placement of large wood. We only considered projects for which the length of the restored reach was a minimum of 200 m; this criterion ensures that the extent of the projects was sufficient to potentially enhance fish assemblages. The mean length of the 18 selected restored reaches was 1.2±1.1 km (± SD). The time between the implementation of the restoration measures and the monitoring in 2007 and 2008 was in the range of 1 to 19 years.

**Table 1:** Overview of the 18 restoration projects.

Project	Parameters						Goals			Measures								
Project No.	Size of catchment (km^2^)	Stream order	Restored section length (km)	Time since restoration (a)	Dispersal obstacles within 5 km	Sampled reaches in species pool	Increase of physical heterogeneity	Flood prevention	Longitudinal connectivity	Lowering of entrenchment depth	Removal of bank fixation	Wood placement	Installation of flow deflectors	Elongation of river length	Creating a new water course	Creation of multiple channels	Extensification of landuse	Re-connection of back waters
1	314	5	0.8	2	56	13	x	–	x	–	–	x	–	–	x	–	x	–
2	2375	7	1.0	2	11	20	x	x	–	–	–	–	–	–	x	x	–	x
3	1290	6	2.0	2	13	27	x	–	–	–	–	–	–	–	–	–	–	x
4	154	5	1.2	1	20	24	x	–	x	–	x	x	–	x	x	–	x	–
5	288	4	0.2	6	18	3	x	–	–	–	x	–	–	x	x	x	–	–
6	278	4	0.3	7	21	6	x	–	x	–	x	–	–	x	x	x	–	–
7	1200	5	0.5	6	15	6	x	–	–	x	x	–	x	x	x	–	x	–
8	1168	5	1.5	1	22	10	x	x	x	x	x	x	x	–	–	x	x	–
9	153	4	0.3	5	18	11	x	–	–	–	x	–	x	–	x	x	x	–
10	658	5	0.8	7	7	12	x	x	–	–	x	–	–	x	x	–	x	–
11	71	3	2.0	5	23	6	x	x	x	x	x	x	x	x	x	x	–	x
12	1000	6	0.8	3	8	11	x	x	–	x	x	x	x	–	–	x	x	–
13	1531	6	0.3	19	15	26	x	–	–	–	–	–	–	x	–	–	x	–
14	90	3	0.5	2	17	10	x	–	–	–	x	–	–	x	x	x	–	–
15	1340	5	0.2	9	14	9	x	–	–	–	x	–	–	–	x	x	x	–
16	116	4	2.6	12	17	6	x	–	–	–	x	–	–	x	x	–	x	–
17	250	4	4.3	10	7	11	x	x	x	–	x	–	–	x	x	–	x	–
18	251	3	0.4	1	30	5	x	–	–	x	x	–	–	x	x	x	–	–
**Mean**	**696**	**5**	**1.1**	**6**	**18**	**12**												
**SD**	**661**	**1**	**1.1**	**5**	**11**	**7**												

An almost identical set of river restoration projects was analysed in Stoll et al. [10]. Only one restoration project was excluded due to lack of quantitative fish assemblage data in the surroundings and replaced by the newly obtained restoration project No. 18 (Table 1). Comparing the fish assemblages at restored and unrestored control reaches, this latter study found a small, however significant positive effect of the restorations on the naturalness of the local fish assemblages. Assemblages at restored sites comprised on average 2.8±1.8 additional species that were part of the stream-type specific natural reference lists compared to the unrestored control reaches, whereas at the same time only 1.3±1.5 of such species were lost [10].

### Monitoring of Hydromorphological Conditions

The local hydromorphological conditions in restored and unrestored control reaches were assessed according to the German hydromorphological survey method [26]; this method is also described by Kamp et al. [27] and Kail and Hering [28]. Twenty-five parameters of the six main groups (planform, longitudinal profile, bed structure, cross-section, bank structure and floodplain corridor) were assessed at each site with a scoring system ranging from 1 (natural) to 7 (completely altered). For further analyses, averaged values for each of the six main groups were used.

Analyses of the hydromorphological data from both restored and unrestored control reaches showed that the restorations significantly improved the hydromorphological conditions [10]. In all six main parameter groups, the restoration projects achieved hydromorphological conditions that can be expected from successful restorations [10], [27]. The hydromorphological conditions in all six main parameter groups were rated similarly, with an average rating around quality level 3 ‘moderate alteration’. Bed structure of restored reaches was rated best; however, on average bed structure also showed the least deficits in unrestored conditions. Greatest improvements as a result of the restorations were achieved in river planform, where on average restored reaches rated 2.6 quality classes better than unrestored reaches.

### Monitoring of Fish

The restored reaches were electrofished in August and September of 2007 and 2008 following the EU Water Framework Directive compliant protocol for the assessment of river fish assemblages in Germany [29], [30]. According to this protocol, wadeable streams were sampled by electrofishing on foot over section lengths of approximately 40× stream width. In non-wadeable streams, electrofishing was executed from a boat. When fishing by boat, sections of a length of approximately 100× stream width were sampled to compensate for the decreased sampling efficiency. The sections were never shorter than 100 m and contained representative proportions of all habitat types present in a reach. Electrofishing was conducted against the current as single passes with generator-powered DC electric fishing gear. Fishing was conducted at stable, low-flow conditions, and extreme discharge events or other adverse conditions were avoided. All stunned fish were placed in trays until the end of fishing, counted and released.

Electrofishing permits for this project were obtained from the Regierungspräsidien Darmstadt, Gießen and Kassel in Hesse and the Untere Landschaftsbehörden in North Rhine-Westphalia. Private land owners were kind enough to provide access to the sampled river reaches. Also protected species were sampled, however not harmed, as all specimens were released at the end of the sampling procedure.

### Data from the Regional Species Pool

The spatial extent of the relevant species pool for the colonization of restored reaches in Central Europe was analysed by Stoll *et al.* [10]. They demonstrated that virtually all fish species that colonized the restored reaches were present in source populations within a distance of 5 km up- or downstream, whereas species for which the closest nearby population was more distant than 5 km rarely colonized restored reaches. Based on these findings, the fish populations in the river network 5 km up- and downstream (including tributaries) were considered to be potential source populations constituting the relevant regional species pool for colonization of the restored reaches. The species compositions of these regional species pools were analysed based on electrofishing data gathered by governmental environmental agencies of the German federal states of Hesse and North Rhine-Westphalia from 1998 to 2008. Electrofishing data from a total of 320 reaches were available and represented 35 different species. On average, 7.2 species occurred per sampled reach. The mean length of the sampled reaches was 257 m (±290 SD). Electrofishing in these surrounding reaches was performed in the same way as in the restored reaches.

### Fish Species Traits

The ecological species traits of habitat preference, feeding type, flow preference and migratory ability were assigned to all present species according to the trait database ‘www.freshwaterecology.info’ [31]. For statistical reasons, individual traits that occurred in fewer than three fish species were pooled for the analyses. Specifically, pelagic habitat preference was pooled with bentho-pelagic habitat preference, in contrast to demersal habitat preference. Furthermore, the feeding types herbivorous, piscivorous and filter-feeding were pooled as ‘specialist’ feeding types because these types all rely on only one food source, in contrast to inverti-piscivorous and omnivorous fish.

Mobility of the species was estimated from the trait swimming factor (SF), which is defined as the aspect ratio of the minimum depth of the caudal peduncle and the maximum caudal fin depth. Fish with a small ratio are capable of strong, sustained swimming [32]. In the www.freshwaterecology.info database, fish species are assigned to three swimming factor categories, small (SF1), medium (SF2) and large (SF3).

Additionally, the species were taxonomically classified to the order level. The orders of perciform, salmoniform and cypriniform fish were differentiated; species from orders with fewer than three present species were clustered as ‘other’.

All species trait information is presented in the Supporting Information Table S1.

### Statistical Analysis

The composition of the regional species pools was characterized using two indices, species occurrence rate and species density. The occurrence rate of a species within a species pool was calculated as the fraction of reaches in which this species was present out of the total number of sampled reaches within that species pool, corresponding to ‘per cent occupancy’ in the study by Albanese et al. [25]. Species density was calculated as the average density of all known occurrences of this species within the species pool.

All statistical analyses were performed in *R* 2.9.1 [33]. In all analyses, each species at each restored reach was regarded as an independent replicate. To analyse the species presence-absence data from the restored reaches, generalized linear models (GLM) for binomial data were used. At each restored reach, present species were assigned the value ‘1’, species that were not present at restored reaches but in the respective regional species pool were assigned ‘0’. Species without proven occurrence in a regional species pool were excluded from the analysis. Two models were used to analyse fish presence-absence data. The first model used ‘biotic’ variables as independent predictors, namely occurrence rate, fish density, taxonomic affiliation, feeding type, habitat preference, flow preference and migratory ability. In the second model, the effects of the ‘abiotic’ variables on species presence at restored reaches were tested. These ‘abiotic’ variables included catchment size, stream order, length of restored reach, time since restoration, planform, longitudinal profile, bed structure, cross-section, bank structure and floodplain corridor. All significant variables from the ‘biotic’ and ‘abiotic’ model were thereafter combined into one model.

Linear models (LM) were used to analyse species densities in the restored reaches. Again two models were fitted using the same independent variables as the GLM models on species presence-absence data. To obtain normal distributions, the species densities in the restored reaches and in the species pools were ln(x+1)-transformed. Again, all significant variables from the ‘biotic’ and ‘abiotic’ model were subsequently combined into one model.

All analyses were initiated with a model containing all variables and second-degree interactions. The models were backward-selected until the minimal Akaike Information Criterion (AIC) was reached.

Our approach to consider each species at each restoration project as an independent replicate may be considered as a potentially pseudo-replicative structure of the dataset. Nevertheless, we chose this approach as the individual colonization events are the basic entities that we were interested in. To consider potential effects involved with pseudo-replication of this approach, a restoration project identifier was added to each model as an additional independent variable; however, this identifier was excluded from the models during the backwards selection procedure. This also points to the broad applicability of the results.

### Data Deposition

Data on regional species pools belonged to the environmental agencies Hessisches Landesamt für Umwelt und Geologie, Hessen-Forst, and Landesamt für Natur, Umwelt und Verbraucherschutz NRW. These data may be requested from the above-mentioned agencies directly. A summary of our own sampling data is published in an electronic appendix to Stoll et al. [10].

## Results

The ‘biotic’ variables, relating to the species composition of the regional species pools and ecological species traits, were much better suited to explain the species assemblages in restored reaches than were the ‘abiotic’ variables, characterizing the restoration projects and local hydromorphological conditions of the restored reaches. The best model using ‘biotic’ descriptors explained 34% of the variability in the species presence, while the best model using ‘abiotic’ descriptors explained only a marginal share of 2% (Table 2a, 3a). In terms of variability in fish densities within restored reaches, the best model using ‘biotic’ descriptors explained 38%, while the best model using ‘abiotic’ descriptors only explained about half of that share, i.e. 21% (Table 2b, 3b). Combining the significant ‘biotic’ and ‘abiotic’ variables into one model did not increase the share of explained variability in species presence data beyond 34%, but for species density data, the share of explained variability increased to 57%. In these combined models, all significant variables from the individual models were retained.

**Table 2:** Best GLM (a, species presence) and LM (b, species density) for the establishment of fish species at a restored site; information on the regional species pool and ecological species traits were used as predictor variables.

	Species occurrence in a restored reach					
	a) Species presence			b) Species density		
AIC	221.01			210.78		
Explained deviance/R^2^_adj_	0.34			0.38		
**Variable**	**Estimate ± SE**	**z**	**P**	**Estimate ± SE**	**t**	**P**
(Intercept)	−1.06±0.39	−2.70	**0.007**	−0.23±0.69	−0.33	0.74
Occurrence rate in RSP	3.08±0.85	3.62	**<0.001**	1.11±0.52	2.12	**0.035**
Density in RSP	0.00±0.00	1.85	0.065	0.45±0.08	5.54	**<0.001**
Habitat preference: demersal				0.54±0.37	1.46	0.15
Order: Perciformes	0.31±0.55	0.56	0.57	−0.19±0.58	−0.33	0.74
Order: Salmoniformes	−2.12±0.59	−3.61	**<0.001**	0.20±0.53	0.39	0.70
Order: others	0.80±0.50	1.60	0.11	−0.90±0.35	−2.56	**0.011**
Flow preference: rheophilic	1.68±0.52	3.24	**0.001**			
Flow preference: limnophilic	−0.73±0.76	−0.96	0.34			
Feeding type: invertivorous				1.45±0.49	2.94	**0.004**
Feeding type: omnivorous				1.41±0.61	2.32	**0.021**
Feeding type: specialists				0.97±0.65	1.49	0.14

The models were backward-selected to the minimum Akaike Information Criterion (AIC). RSP = regional species pool.

**Table 3:** Best GLM (a, species presence) and LM (b, species density) for the establishment of fish species at a restored site; information on local hydromorphological conditions, the restoration projects and the rivers in which the projects were undertaken were used as predictor variables.

	Species occurrence in a restored reach					
	a) Species presence			b) Species density		
AIC	197.76			234.73		
Explained deviance/R^2^_adj_	0.02			0.21		
**Variable**	**Estimate ± SE**	**z**	**P**	**Estimate ± SE**	**t**	**P**
(Intercept)	−0.65±0.74	−0.87	0.38	8.96±0.90	9.96	**<0.001**
Cross-section profile	0.33±0.20	1.65	0.09			
Time	0.10±0.04	2.59	**0.009**			
Planform				−0.38±0.15	−2.46	**0.015**
Floodplain				−0.65±0.12	−5.44	**<0.001**
Stream order				−0.33±0.14	−2.41	**0.017**

The models were backward-selected to the minimum Akaike Information Criterion (AIC).

**Figure 1:**
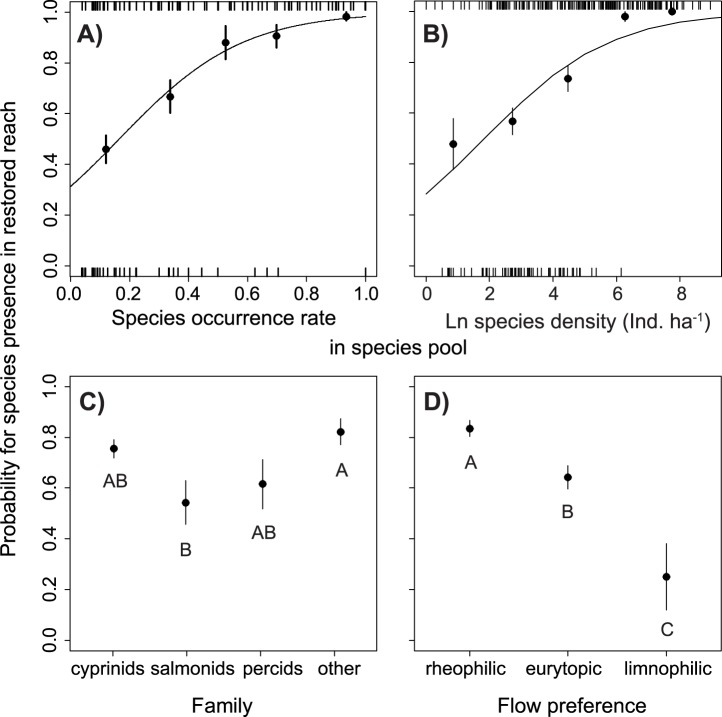
The probability of a species presence (± SE) in restored reaches as a function of (A) their occurrence rates and (B) species densities in the regional species pool. Differences in the probability that a species is present in the restored reach are shown with regard to (C) taxonomic affiliation and (D) flow preferences. The tick marks on the axes in (A) and (B) indicate the individual data points to which the logistic models (curve) were fitted. As a visual aid to estimate of the fit of the logistic models in (A) and (B), empirical probabilities (±SE) of five evenly spaced sectors of the data were added (dots and error bars). Letters in (C) and (D) indicate homogenous groups according to Tukey HSD post-hoc tests.

The presence of a species within the restored reaches was particularly dependent on the occurrence rate of the species in the species pool. For example, species that occurred at 10% of the sampling sites in a regional species pool showed an average probability of 45% to colonize the restored reach of that river (Figure 1a). Species with occurrence rates in the regional species pool greater than 75% colonized every restored reach. The density of a species within the regional species pool had a marginally significant effect on the presence of that species in restored reaches. Species with a density of about 7 ind. ha^−1^ showed a probability of about 50% to colonize a restored reach. All species with average densities greater than 470 ind. ha^−1^ in a regional species pool colonized the respective restored reach (Figure 1b).

Salmonid species, including brown trout (*Salmo trutta*), rainbow trout (*Oncorhynchus mykiss*), Atlantic salmon (*Salmo salar*) and grayling (*Thymallus thymallus*) exhibited the lowest probabilities of colonizing restored reaches, while fish species that belonged to the mixed group labelled ‘other’, including eel (*Anguilla anguilla*), brook lamprey (*Lampetra planeri*), pike (*Esox lucius*), three-spined stickleback (*Gasterosteus aculeatus*), ten-spined stickleback (*Pungitius pungitius*) and bullhead (*Cottus gobio*), showed the highest probabilities of emerging in restored reaches (Figure 1c). Rheophilic species exhibited a higher probability of colonizing restored reaches than did limnophilic species (Figure 1d); eurytopic species that are indifferent to flow conditions showed an intermediate response.

**Figure 2:**
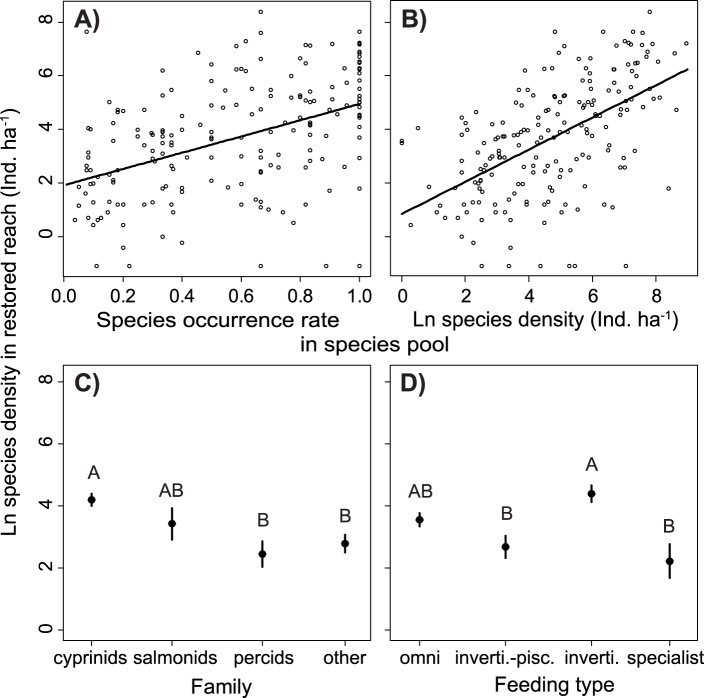
Species density (± SE) in restored reaches as a function of (A) species occurrence rates and (B) the average density of a species in the regional species pool. The differences in species densities in the restored reaches are shown with respect to (C) taxonomic affiliation and (D) flow preferences of the species. Letters in (C) and (D) indicate homogenous groups according to Tukey HSD post-hoc tests.

Higher density of a species within a restored reach was related to a combination of high occurrence rate and the density of that species in the regional species pool (Figures 2a, b). Cyprinids exhibited the highest densities in restored reaches, e.g. minnow (*Phoxinus phoxinus*) 1022±547 ind. ha^−1^, stone loach (*Barbatula barbatula*) 676±147 ind. ha^−1^ and gudgeon (*Gobio gobio*) 463±141 ind. ha^−1^(all mean ± SD). Percids and ‘other’ species occurred at the lowest densities, e.g. pike 10±4 ind. ha^−1^, eel 6±2 ind. ha^−1^ and pikeperch (*Sander lucioperca*) 12±11 ind. ha^−1^ (Figure 2c). Densities in restored reaches also differed between feeding types. Species that consume fish as some part of their diet occurred at low densities in restored reaches, while invertivorous species exhibited the highest densities in restored reaches (Figure 2d).

**Figure 3:**
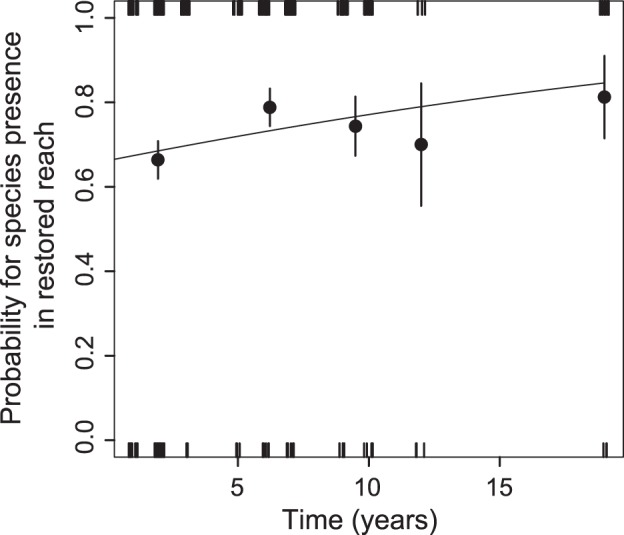
The probability of a species to be present in restored reaches as a function of the time lag between restoration and sampling. The tick marks on the axes indicate the individual data points to which the logistic model is fitted. As a visual aid to estimate of the fit of the logistic model, empirical probabilities (±SE) of five sectors of the data were added (dots and error bars).

**Figure 4:**
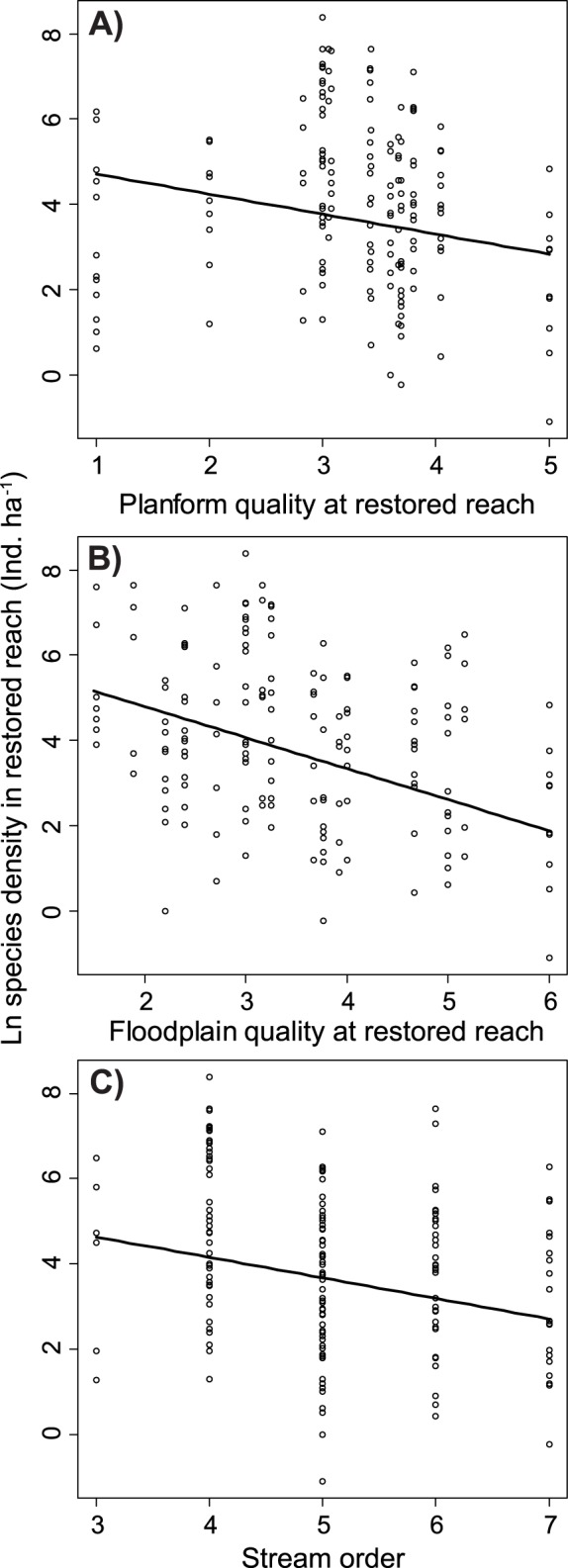
Species density (± SE) in restored reaches as a function of the hydromorphological quality metrics (A) planform quality and (B) floodplain quality according to the river habitat assessment scheme developed by Kamp et al. [**27**] **.** The scale of these metrics spans from 1 (natural conditions) to 7 (completely altered conditions). (C) Dependence of species density on the Strahler stream order.

Among the set of ‘abiotic’ variables, the presence of a species in a restored reach depended only on the time between restoration and sampling. Within the first year after the restoration work was completed, the probability of a species being present in a restored reach was about 65%; this percentage further increased with the time elapsed since the restoration work was performed, to about 80% after 19 years (Figure 3). A positive influence of two hydromorphological quality metrics, planform and floodplain quality, on the species density in restored reaches was detected (Figures 4a, b), and higher fish densities were found in reaches of lower stream order (Figure 4c).

## Discussion

Our study showed that, in total, 57% of the variability in the fish density data and 34% of the variability in the fish presence-absence data at restored reaches is explained by a set of simple ‘biotic’ and ‘abiotic’ variables. Strikingly, ‘biotic’ variables characterizing the regional species pools were much better suited to explain fish presences and abundances in the restored reaches than ‘abiotic’ variables characterizing the restoration projects and hydromorphological structures in restored reaches. A particular importance of the regional species pools for river restoration outcomes has already been assumed, but not quantitatively demonstrated [10], [20].

The river restoration projects in this study, in line with other studies on reach-scale river restoration projects, were successful in removing hydromorphological limitations and providing natural (or at least near-natural) habitat conditions [6], [10], [34]. So if species do not colonize such restored reaches, it is more likely that this is because of absence or rarity in the regional species pools and limitations in the dispersal process than because of lack of habitat suitability at a restored reach.

The most important individual variable for the probability of species to colonize a restored river reach was the occurrence rate of this species within the regional species pool, while high population densities in surrounding areas were more important for the size of a population in a restored reach. Large populations provide not only a higher number of emigrants when a fixed rate of individuals is expected to emigrate but may also have an overall higher rate of emigrants to avoid intraspecific competition [35]. Using a modelling approach, already Huxel and Hastings [36] postulated that species establishment at restored reaches depends on habitat occupancy of these species in neighbouring reaches. In a fish removal experiment, Albanese et al. [25] confirmed the role of species abundance in the regional species pool for the colonization of emptied reaches, and further highlighted the role of fish mobility in colonizing such reaches. Also Radinger and Wolter [24] found a relation between fish mobility and dispersal distance of fish species, whereat they also estimated a species mobility from a species swimming factor [31]. In the present study, fish mobility was not retained in the best models explaining the fish assemblages in the restored reaches. Nonetheless, there is a considerable overlap between the ecological traits, mobility and rheophily, as many rheophilic species are strong swimmers [37]. However, some rheophilic species, despite living in riffle or run environments avoid fast flow by choosing appropriate micro-habitats in the interstices of the bottom substratum [38], e.g. bullhead. Inversely, our results suggest that stretches of riffles in river networks may pose considerable dispersal impediments, particularly for limnophilic species [39]. Furthermore, rheophilic species may be generally more avid dispersers as they have to be able to compensate for drift and it has been shown for a number of species that peak flow triggers upstream movement [40].

An alternative interpretation of the higher probability of rheophilic species to be present at restored sites is that the newly created habitats are more suitable for such species. However, this appears relatively unlikely because the restorations did not increase flow velocities, but as a result of river widening, elongation of river courses and reconnection of backwaters, the variability of flow velocities is significantly increased [34]. Thus, additional habitats for both rheophilic and limnophilic species were created. This evidences that the mechanism behind the different colonization success between rheophilic and limnophilic species is rather connected with dispersal than with habitat suitability of restored reaches.

In addition to the quantitative metrics of the regional species pools, taxonomic affiliation, i.e. species identities, had an effect both on the probability to colonize a restored reach and on the resulting species densities. Salmonids showed a sub-average probability to colonize restored reaches, particularly compared to species of ‘other’ orders. Among these latter species, brook lamprey, three-spined sticklebacks and bullhead, in particular, are known from other studies to be strong colonizers [10]. The result that the heterogeneous ‘other’ group showed the highest colonization probabilities makes it difficult to reveal the relevant underlying ecological traits. Nevertheless, even though being successful colonizers, these ‘other’ species did not build up high densities at restored reaches. Instead, cyprinids showed almost as high colonization probabilities and additionally were most likely to reach high densities in restored reaches. Most cyprinid species are gregarious [41], and fish density in the regional species pool has been revealed as a major factor for the density of a species at a restored reach. Furthermore, as cyprinids typically feed on lower trophic levels, habitats support higher fish densities than in taxonomic groups that predominantly forage at the upper end of the food chain, such as for example percids that occur only at lower densities.

Among the ‘abiotic’ predictors of species presence at restored reaches, time since the restoration was carried out was most important. It is often assumed that, on small spatial scales, the colonization process will proceed rapidly [42]. However, after this initial colonization by nearby species, colonization by species through long-distance dispersal has been shown to be a slow and highly stochastic process [10], [43], [44]. Because datasets are often small and comprise only data of one or few restoration projects, it is difficult to demonstrate rare colonization events following long-distance dispersal; as such incidents may be masked by the natural species turnover within local fish assemblages or by the limitations in the detectability of small populations by electrofishing.

None of the six metrics of river hydromorphological quality affected the probability of a species to colonize a restored reach; however, fish densities at restored reaches were positively influenced by river planform and floodplain quality. Planform is a good proxy for the overall hydromorphological state [28] because high sinuosity and braidedness is usually associated with high habitat diversity (e.g., bars, pools and undercut banks) and lateral channel dynamics. Dynamic changes in a river course due to relocating gravel bars, erosion and deposition provide essential habitat for many riverine species, especially as nursery areas [14], [45], [46], and emerging shallow water areas reduce current velocity and provide shelter from predatory fish [47], [48]. Also good rating results of the hydromorphological parameters related to floodplain quality, comprising information on riparian features including provision of shading and land use across the entire floodplain, is often associated with high fish densities. For instance, fish often aggregate under riparian structures providing protection from aerial predators [49]. Also differences in land use affect fish, albeit on larger spatial scales than the effects of shading [50], [51], leading to impoverished fish fauna and lower fish densities in intensely used systems [52].

In small, low-order rivers, the fish densities in restored reaches were higher than in large high-order rivers. On a per-area basis, fish abundance is often higher in small and medium-sized rivers compared to large rivers, as the former typically provide more diverse and more complex habitat structures, which typically aggregate fish [53], [54]. Part of the difference in fish densities between restored reaches in low- and high-order rivers may also be explained by the decreasing effectiveness of electrofishing with increasing river size [55].

### Implications for Restoration Planning

This study demonstrates that ‘biotic’ data on the regional species pools may be used to estimate the probability of fish species to colonize a restored reach. These ‘biotic’ data are much better suited to explain fish presence and densities at restored reaches than ‘abiotic’ data, which are often used as the base for such attempts in other studies. The results of this study highlight the paramount importance of appropriate spatial prioritization in river restoration planning. Only if the regional species pools are intact and diverse, will the removal of hydromorphological deficits succeed in enhancing the naturalness of local fish assemblages. Therefore, in addition to focusing on local hydromorphological conditions and engineering aspects, the regional species pools should receive more attention when planning river restoration projects. In the prioritization of alternative restoration sites, knowledge on the status of the regional species pool permits estimation of the likelihood of restoration projects to reach specific targets. Only species with a sufficient abundance in a regional species pool can be expected to colonize a restored reach, whereat the critical values of individual species may vary, depending on their ecological traits.

Species with low abundance in the regional species pool are unlikely to colonize restored reaches. Thus, restoration projects aiming to improve general habitat structure have little chance of success in supporting endangered species, which typically have small and scattered populations. In cases where restorations are designed to support individual species (e.g. endangered species with particular habitat needs) a distinct focus on the limiting habitat features may be more promising. To support such species with scarce or only fragmented source populations, stocking, sometimes also referred to as assisted migration, may be an option; however, this practice has been heavily debated in recent years [56]–[58].

## Supporting Information

Table S1 List of the 35 fish and lamprey species that occurred in the samples collected in the restored reaches and their relevant 5-km species pools as well as ecological traits of these species.
(DOC)
