# Supplementary material for: The Importance of the Regional Species Pool, Ecological Species Traits and Local Habitat Conditions for the Colonization of Restored River Reaches by Fish
Source: PLoS One. 2014 Jan 3;9(1):e84741. doi: 10.1371/journal.pone.0084741 (PMC3880337; doi:10.1371/journal.pone.0084741)
Supplement: Table S1 — List of the 35 fish and lamprey species that occurred in the samples collected in the restored reaches and their relevant 5-km species pools as well as ecological traits of these species. (DOC) [file pone.0084741.s001.doc]

**Supporting Information**

**Table S1**: List of the 35 fish and lamprey species that occurred in the samples collected in the restored reaches and their relevant 5-km species pools. The ecological species traits habitat preference, flow preference, swimming factor and feeding type, and were assigned to all occurring species according to the trait database ‘freshwaterecology.info’ [1].

| **Species name** | |  | **Species presence** | | **Taxonomic affiliation** | **Habitat preference** | **Flow preference** | **Swimming factor** | **Feeding type** |
| --- | --- | --- | --- | --- | --- | --- | --- | --- | --- |
| **English** | **Latin** |  | **N species pools** | **N restored reaches** |
| Atlantic salmon | *Salmo salar* |  | 4 | 1 | Salmoniformes | bentho-pelagic | rheophilic | 2 | invertivorous |
| barbel | *Barbus barbus* |  | 9 | 9 | Cypriniformes | bentho-pelagic | rheophilic | 1 | invertivorous |
| bitterling | *Rhodeus amarus* |  | 5 | 4 | Cypriniformes | bentho-pelagic | eurytopic | 1 | omnivorous |
| bleak | *Alburnus alburnus* |  | 1 | 1 | Cypriniformes | bentho-pelagic | eurytopic | 1 | omnivorous |
| bream | *Abramis brama* |  | 6 | 1 | Cypriniformes | bentho-pelagic | eurytopic | 1 | omnivorous |
| brook lamprey | *Lampetra planeri* |  | 5 | 5 | other | demersal | rheophilic | 3 | filter-feeding -> specialist |
| brown trout | *Salmo trutta m. fario* |  | 15 | 10 | Salmoniformes | demersal | rheophilic | 2 | inverti-piscivorous |
| bullhead | *Cottus gobio* |  | 10 | 9 | other | demersal | rheophilic | 2 | invertivorous |
| carp | *Cyprinus carpio* |  | 4 | 1 | Cypriniformes | bentho-pelagic | eurytopic | 2 | omnivorous |
| chub | *Leuciscus cephalus* |  | 17 | 17 | Cypriniformes | bentho-pelagic | rheophilic | 2 | omnivorous |
| Crucian carp | *Carassius carassius* |  | 2 | 0 | Cypriniformes | demersal | limnophilic | 3 | omnivorous |
| dace | *Leuciscus leuciscus* |  | 15 | 14 | Cypriniformes | bentho-pelagic | rheophilic | 2 | omnivorous |
| eel | *Anguilla anguilla* |  | 16 | 11 | other | demersal | eurytopic | 3 | inverti-piscivorous |
| grayling | *Thymallus thymallus* |  | 8 | 6 | Salmoniformes | bentho-pelagic | rheophilic | 2 | invertivorous |
| gudgeon | *Gobio gobio* |  | 17 | 17 | Cypriniformes | bentho-pelagic | rheophilic | 1 | invertivorous |
| ide | *Leuciscus idus* |  | 4 | 0 | Cypriniformes | bentho-pelagic | rheophilic | 2 | omnivorous |
| minnow | *Phoxinus phoxinus* |  | 9 | 9 | Cypriniformes | demersal | rheophilic | 1 | invertivorous |
| nase | *Chondrostoma nasus* |  | 1 | 0 | Cypriniformes | bentho-pelagic | rheophilic | 1 | herbivorous -> specialist |
| perch | *Perca fluviatilis* |  | 16 | 11 | Perciformes | demersal | eurytopic | 2 | inverti-piscivorous |
| pike | *Esox lucius* |  | 8 | 4 | other | demersal | eurytopic | 2 | piscivorous -> specialist |
| pikeperch | *Sander lucioperca* |  | 3 | 2 | Perciformes | pelagic -> bentho-pelagic | eurytopic | 2 | piscivorous -> specialist |
| Prussian carp | *Carassius gibelio* |  | 3 | 1 | Cypriniformes | bentho-pelagic | eurytopic | 3 | omnivorous |
| pseudorasbora | *Pseudorasbora parva* |  | 8 | 4 | Cypriniformes | bentho-pelagic | eurytopic | 2 | omnivorous |
| pumpkinseed sunfish | *Lepomis gibbosus* |  | 1 | 0 | Perciformes | bentho-pelagic | eurytopic | 3 | invertivorous |
| rainbow trout | *Oncorhynchus mykiss* |  | 8 | 2 | Salmoniformes | bentho-pelagic | rheophilic | 1 | inverti-piscivorous |
| roach | *Rutilus rutilus* |  | 16 | 11 | Cypriniformes | bentho-pelagic | eurytopic | 1 | omnivorous |
| rudd | *Scardinius erythrophthalmus* |  | 3 | 1 | Cypriniformes | bentho-pelagic | limnophilic | 2 | omnivorous |
| ruffe | *Gymnocephalus cernuus* |  | 6 | 3 | Perciformes | demersal | eurytopic | 1 | invertivorous |
| silver bream | *Abramis bjoerkna* |  | 2 | 1 | Cypriniformes | demersal | eurytopic | 1 | omnivorous |
| spined loach | *Cobitis taenia* |  | 1 | 1 | Cypriniformes | bentho-pelagic | rheophilic | 3 | invertivorous |
| stone loach | *Barbatula barbatula* |  | 17 | 17 | Cypriniformes | demersal | rheophilic | 3 | invertivorous |
| sun bleak | *Leucaspius delineatus* |  | 1 | 0 | Cypriniformes | pelagic -> bentho-pelagic | limnophilic | 1 | omnivorous |
| tench | *Tinca tinca* |  | 6 | 2 | Cypriniformes | demersal | limnophilic | 3 | omnivorous |
| ten-spined stickleback | *Pungitius pungitius* |  | 1 | 1 | other | bentho-pelagic | eurytopic | 1 | omnivorous |
| three-spined stickleback | *Gasterosteus aculeatus* |  | 16 | 16 | other | bentho-pelagic | eurytopic | 1 | omnivorous |

1. Grenouillet G, Schmidt-Kloiber A (2006) Fish Indicator Database. Euro-limpacs project (contract no. GOCE-CT-2003-505540), Workpackage 7 - Indicators of ecosystem health, Task 4, www.freshwaterecology.info (version 4.0).
